# Positional bias of general and tissue-specific regulatory motifs in mouse gene promoters

**DOI:** 10.1186/1471-2164-8-459

**Published:** 2007-12-13

**Authors:** Nicolás Bellora, Domènec Farré, M Mar Albà

**Affiliations:** 1 Research Unit on Biomedical Informatics, Universitat Pompeu Fabra, Barcelona, Spain; 2 Centre for Genomic Regulation, Barcelona, Spain; 3 Fundació Institut Municipal d'Investigació Mèdica, Barcelona, Spain; 4 Catalan Institution for Research and Advanced Studies, Barcelona, Spain

## Abstract

**Background:**

The arrangement of regulatory motifs in gene promoters, or promoter architecture, is the result of mutation and selection processes that have operated over many millions of years. In mammals, tissue-specific transcriptional regulation is related to the presence of specific protein-interacting DNA motifs in gene promoters. However, little is known about the relative location and spacing of these motifs. To fill this gap, we have performed a systematic search for motifs that show significant bias at specific promoter locations in a large collection of housekeeping and tissue-specific genes.

**Results:**

We observe that promoters driving housekeeping gene expression are enriched in particular motifs with strong positional bias, such as YY1, which are of little relevance in promoters driving tissue-specific expression. We also identify a large number of motifs that show positional bias in genes expressed in a highly tissue-specific manner. They include well-known tissue-specific motifs, such as HNF1 and HNF4 motifs in liver, kidney and small intestine, or RFX motifs in testis, as well as many potentially novel regulatory motifs. Based on this analysis, we provide predictions for 559 tissue-specific motifs in mouse gene promoters.

**Conclusion:**

The study shows that motif positional bias is an important feature of mammalian proximal promoters and that it affects both general and tissue-specific motifs. Motif positional constraints define very distinct promoter architectures depending on breadth of expression and type of tissue.

## Background

The control of gene transcription is mediated by transcription factors, which interact in a sequence-specific manner with DNA motifs, known as transcription factor binding sites (TFBS). These motifs are abundant in gene promoter regions, upstream from the transcription start site (TSS). The promoter is often divided into the basal or core promoter, covering approximately 100 bp upstream of the TSS, and the proximal promoter, which extends up to a few hundred base pairs and typically contains multiple sites for activators [1]. Other functional regions, such as enhancers, can be found at very distant locations from the TSS. However, it appears that the region spanning from -550 to +50 with respect to the TSS is sufficient, in a large proportion of human genes, to drive transcriptional activity in cultured cells [2].

One important aspect of promoter sequences is the specific arrangement of regulatory motifs along the DNA sequence, and the existence of recurrent patterns in the relative position of motifs. It has been observed that a number of TFBS, including motifs for some of the most abundant transcription factors, show a tendency to cluster in the proximal promoter [3-7]. For example CCAAT enhancer binding protein (CEBP) motifs are basically found within an area from -100 to -50 with respect to the TSS [8]. Another example is cyclic-AMP response element (CRE), found in mammals far more frequently within 150 bp upstream from the TSS than in any other region [9]. On the other hand, it has been recently observed that a number of motifs that are likely to be important for the regulation of the expression of ribosomal protein genes are located at fixed positions within the promoter [10]. It is also well-known that TFBS can be arranged in particular combinations forming functional regulatory units, known as *cis*-regulatory modules [11,12]. Spacing between motifs can be the result of transcription factor interaction requirements in the context of particular *cis*-regulatory modules. This type of constraints can be revealed by the analysis of relative motif positions in many different genes, with the discovery of recurrent motif location patterns or 'positional footprints'. A tool that can be used to detect motif frequency profiles, using DNA words or a restricted set of known TFBS matrices, is Signal search analysis server [13]. We have recently developed another application, PEAKS [14,15], which, in addition to oligomers, uses existing TFBS matrix libraries, calculates 'positional footprinting' scores and associated p-values, and produces integrated motif views from large gene datasets. Here we use PEAKS to perform the most exhaustive to date analysis of motif positional biases in mammalian gene promoters. To explore the effect of tissue-specificity we use microarray data from 55 mouse tissues [16]. The analysis identifies distinctive features of promoters driving housekeeping or tissue-specific expression, shows that a number of well-known tissue-specific regulatory motifs are subject to strong positional constraints and predicts novel regulatory elements in different tissue expression gene datasets.

## Results

### Positional bias of general motifs

**Figure 1:**
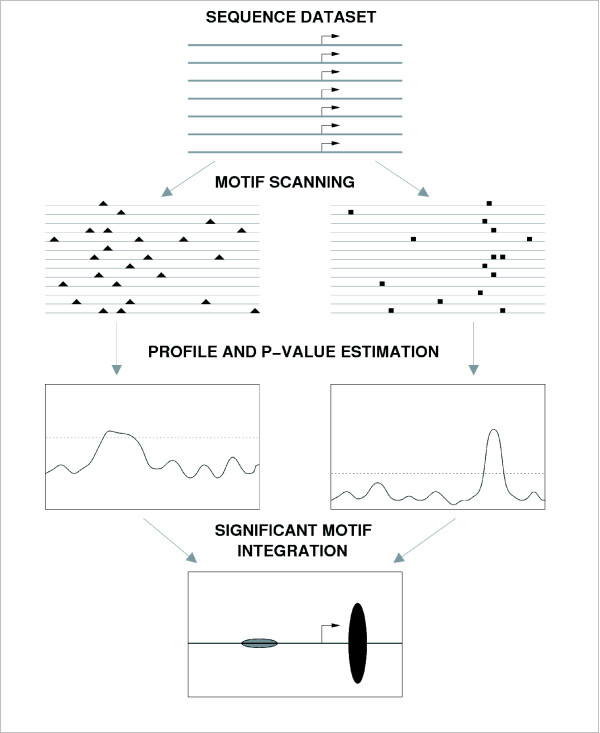
**Schematic representation of the PEAKS method**. Detection of positional bias of two hypothetical motifs in a promoter sequence dataset is shown. After motif scanning, a profile of motif frequency is obtained. The horizontal line delineates the region above a given p-value cut-off. Significant regions are plotted into a single integrated representation.

We collected mouse gene sequences, spanning from -600 to +100 with respect to the TSS, using the UCSC genome database [17]. This is what we will term "promoters", although it approximately corresponds to what is generally understood as the proximal promoter region. In the first place, we aimed at identifying general motifs that showed a positional bias in a significant number of promoters. We analyzed 6,372 non-redundant mouse gene promoter sequences with the previously developed program PEAKS [14,15]. A scheme of the procedure employed by PEAKS is shown in Figure 1. The first step is the identification of putative motifs on the sequences using one or more motif libraries. In this study we used four different libraries: 508 vertebrate weight matrices corresponding to known transcription factor binding sites from TRANSFAC [18]; 91 vertebrate weight matrices corresponding to known transcription factor binding sites from JASPAR, or JASPAR CORE matrices [19]; 174 weight matrices from JASPAR corresponding to putative regulatory sequences on the basis of phylogenetic conservation, or JASPAR phyloFACTS [19]; and a non-redundant set of 2080 oligomers of size 6 (6mers). The second step of the procedure is the generation of motif frequency profiles along the promoter. The profiles represent the number of sequences in which a motif is predicted at least once in a sequence window surrounding each position. In this analysis, we used a window size of 31 nucleotides, so occurrence of motifs anywhere from -15 to +15 with respect to the central position was sufficient for that position to be positive. The use of sliding windows, instead of strict positions, provides a certain degree of flexibility to accommodate functional motif and TSS position variability. The third step is the calculation of the positional footprinting score (*Spf*) of the position with the highest motif frequency (maximum peak in the profile). This score measures the tendency of the motif to be located in a particular region of the promoter, taking into account its overall abundance and distribution [14]. Using random sequences that mimic nucleotide variability along promoters, we obtain the p-value that corresponds to any particular *Spf *score. Promoter sequences contain regions with very biased GC content. To model realistic sequence datasets we first partition all mouse promoter sequences into three distinct types of regions according to their composition: 1. CpG islands; 2. GC-rich regions that are not CpG islands and; 3. The rest of regions (see Methods for an exact definition). We derive three distinct order 1 Markov chain models from sequence regions that belong to the same compositional class. Using these Markov chains, we generate random sequence datasets with the same number of sequences, and same partitioning in region types, as in the real sequences. As a result, the random sequences show similar composition to the real sequences along the promoter (Additional file 1). Throughout this work, we used a p-value <= 10^-5 ^to identify motifs with significant *Spf *scores, unless stated otherwise.

**Figure 2:**
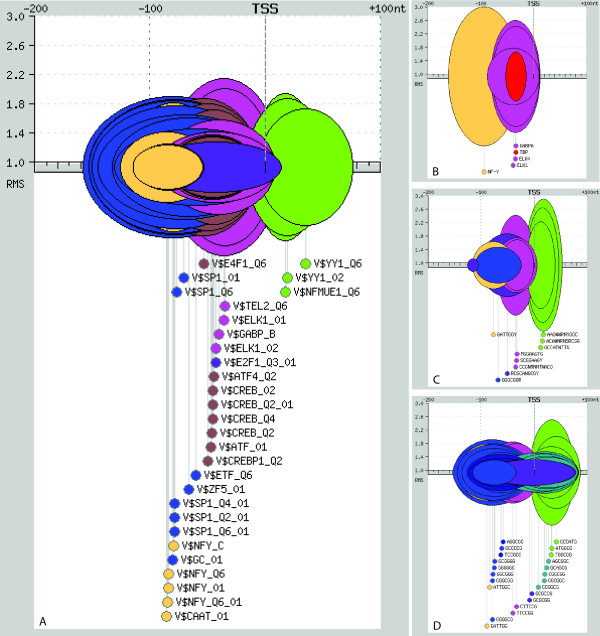
**Integrated representation of motifs with significant positional bias in mouse promoters**. The results were obtained by the program PEAKS, using different motif libaries. A. TRANSFAC PSWMs. B. JASPAR CORE PSWMs. C. JASPAR phyloFACTS. D. oligomers of size 6 (6mers). Motifs that belong to the same motif cluster are shown with the same color. A region from -200 to +100 with respect to the TSS is shown. The width of the ovals is the significant region of each motif (p-value <= 10^-5^). The height of the ovals, the relative motif signal (RMS), is the number of sequences that contain a motif located at the position with the maximum score divided by the minimum number of sequences containing that motif that would be required to pass the p-value cut-off.

In the complete mouse promoter dataset, we identified 29 significant motifs corresponding to matches to TRANSFAC matrices, 4 to JASPAR CORE matrices, 9 to JASPAR phyloFACTS, and 22 to 6mers. In many cases, the same motif was found by several of the libraries, but in other cases the information obtained was complementary. Although we considered a promoter region of length 700 nucleotides (from -600 to +100), all motifs were found in a much smaller region, between around -150 and +100. Basically, they were binding sites for general, commonly found, transcription factors. Figure 2 shows an integrated representation of the significant motifs obtained with the four libraries. To deal with motif redundancy, both within and across libraries, we clustered the motifs on the basis of the degree of overlap in all promoter sequences (see Methods and Additional file 2). We obtained nine different motif clusters, which are plotted with the same color in Figure 2.

**Figure 3:**
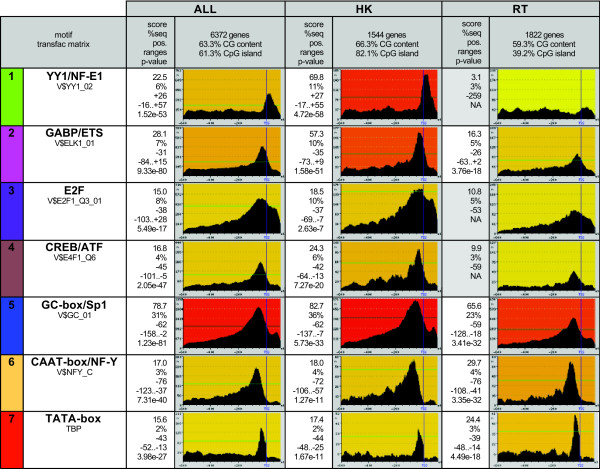
**Promoter motif profiles in mouse genes with different expression width**. ALL: complete promoter dataset; HK: housekeeping genes; RT: genes with restricted expression. Profiles were obtained with the program PEAKS using window size 31. Profiles with no significant sequence ranges (NA) did not accomplish p-value <= 10^-5^. Left-most cells contain the TRANSFAC matrix (or JASPAR for TBP) used for motif prediction and the significant regions in the different datasets. Background color indicates score value grading, from intense red (highest) to pale yellow (lowest). 'score' is the positional footprinting score; '%seq' percentage of sequences at maximum peak; 'pos.', position of the maximum peak; 'ranges' sequence interval significant above the p-value cut-off.

The nine different types of motifs showed characteristic preferential positions with respect to the TSS (Figure 2, Figure 3 ALL, and Additional files 3, 4, 5, 6). The Ying and Yang (YY1/NF-E1) binding site motif was found downstream of the TSS (maximum peak at +26 with the TRANSFAC matrix V$YY1_02). In addition to several TRANSFAC matrices (V$YY1_02, V$YY1_Q6 and V$NFMUE1_Q6), this motif was detected by three different JASPAR phyloFACTS (AAGWWRNYGGC, ACAWNRNSRCGG and GCCATNTTG) and three 6mers (GCCATG, ATGGCG, TGGCGG). Another characteristic element that we detected in this region was made of repeats of GGC or AGC. This motif was only detected with 6mers, and maximum peaks were located between +18 and +28 depending on the specific 6mer (see Figure 2 and Additional file 6). Upstream from the TSS, in a region around -20 to -40, we detected three types of motifs corresponding to known transcription factor binding sites. The first one corresponded to binding sites for the ETS-domain containing family of transcription factors: TEL, ELK and GABP (maximum peak at -31 with V$ELK_01). The second one was the TATA box (maximum peak at -36 with the JASPAR CORE TBP matrix). The third motif was the E2F binding site (maximum peak at -38 with V$E2F1_Q3_Q1). Transcription factors containing the ETS domain are involved in the regulation of transcription in a great variety of biological processes in metazoans [20]. On the other hand, E2F factors have been reported to be important for the control of the cell cycle [21]. Further upstream we found CREB-type motifs (cAMP response element-binding), which are bound by CBP/ATF/E4F transcription factors (maximum peak at -45 with V$E4F1_Q6). The region that was significant for GC-box/SP1 motifs was located further upstream (maximum peak at -62 with V$GC_01). The transcription factor SP1 is involved in the expression of many different genes, and can interact with other transcription factors, such as TBP (TATA-binding protein), Ying and Yang and E2F [22]. Other GC-rich motifs, corresponding to binding sites for factors ZF5 and ETF were part of the same motif cluster. A motif resembling the SP1 motif, but sufficiently distinct to be part of a different cluster, was identified with 6mers AGGCGG and TCCGCC (GGCGGA when reversed), around the same region. Finally, we identified CAAT-box/NF-Y motifs in a more upstream position (maximum peak at -76 with V$NFY_C).

### Widely expressed versus tissue-specific genes

We next classified the mouse promoter sequences in several groups according to where the gene was expressed, using normalized microarray data from 55 different mouse organs and tissues [16]. In the first place we wanted to investigate if the arrangement and nature of the most common motifs depended on the breadth of expression. We defined a group of genes with expression limited to 1–10 tissues ('restricted', 1822 genes) and a second group of genes with expression in 51–55 different tissues ('housekeeping', 1544 genes). A comparison of the results obtained in the three different datasets – complete promoter dataset (ALL), housekeeping genes (HK) and restricted genes (RT) – is shown in Figure 3. In each motif profile, the region of the peak that is above the line is the motif significant region (represented by the width of the oval in Figure 2). Only one representative TRANSFAC or JASPAR matrix per motif cluster is shown, the complete data is available in Additional files 3, 4, 5, 6.

Interestingly, there were very clear differences between HK and RT genes. The peak corresponding to motifs for Ying and Yang and nuclear factor E1 (YY1/NF-E1) was much sharper in housekeeping genes than in the general dataset (compare HK to ALL in Figure 3), and completely absent from genes with restricted expression (RT). In the HK dataset, 11% of the genes contained this motif in position +27, while this number was only 3% for RT genes, around the level of background signal for this motif. The YY1 factor is ubiquitous and involved in the control of basal transcription [23], which is consistent with our results. Besides YY1, two other motifs did not achieve statistical significance in the RT dataset. The first one was the cluster CREB/ATF/E4F, which showed a much sharper peak in the HK dataset than in the RT dataset. In particular, the percentage of HK genes containing E4F motifs at the maximum peak position was 6%, twice that of RT genes. E4F is a ubiquitously expressed protein reported to be important for mitotic progression [24]. The other motif that was not significant in RT genes was the E2F binding site, which was also about twice as frequent in HK genes than in RT genes. On the other hand, GABP/ETS and GC-box/Sp1 motifs also showed higher *Spf *scores in HK than in RT. Contrary to the motifs mentioned above, the TATA-box, as well as CAAT-box/NF-Y, were stronger in the RT dataset than in the HK dataset, although clearly significant in both.

An important outcome of the comparison between ALL, HK and RT datasets was that most of the motifs showed higher scores in the HK dataset than in the RT dataset. This is not surprising, as the latter are an amalgam of genes with very diverse expression patterns, and so they are likely to have more varied motif configurations or more distally located regulatory regions. We also observed that the average GC content, and in special the proportion of genes with CpG islands, was higher in HK than in RT gene promoters (Figure 3). Average GC content was 66.3% in HK and 59.3% in RT, while the number of CpG island-containing promoters was 82% in HK and 39.2% in RT. These differences are in agreement with previous reports [25,26].

### Tissue-specific motifs

We analyzed in greater detail the genes showing strong tissue-specificity, by performing a separate analysis of groups of RT genes expressed in each of the different adult tissues (N = 47). For example the dataset 'liver' was composed of genes from the RT class (expressed in 1–10 tissues) that showed expression in liver. One can expect that some tissues will be more similar to each other, in regard to the genes that they express, than others. To learn about this, we clustered the tissues according to the number of shared expressed genes. We identified four main clusters, in which every pair of tissues shared at least 30% of the genes of one tissue. The clusters, A to D, corresponded, to a large extent, to known physiological systems (Additional file 7). Cluster A was composed by diverse tissues from the nervous system; cluster B was mainly composed by tissues related to the digestive system; cluster C by muscle and skin tissues; and cluster D by bone, lymph and bladder. We obtained non-redundant gene datasets for each cluster. These datasets were composed of RT genes for which at least 50% of the tissues in which they were expressed belonged to that cluster. Surprisingly, commonly found motifs (those shown in Figure 2) showed a very different distribution in different RT gene clusters (Additional data files 3, 4, 5, 6). For example, the GABP/ETS motif, as well as CREB/ATF, only reached significant scores in cluster D; the GC-box/SP1 was only significant in cluster A and B; and, the CAAT-box/NF-Y was only significant in cluster A.

**Figure 4:**
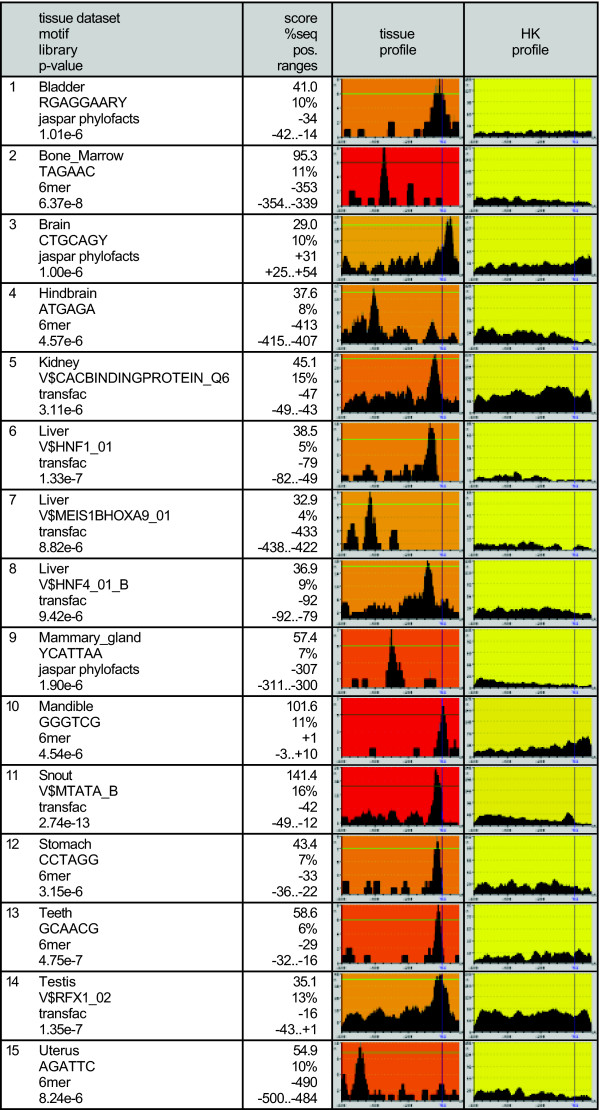
**Promoter motif profiles in mouse genes expressed in particular tissues**. Selection of motifs that were significant in genes expressed in a particular tissue but not in the housekeeping (HK) dataset. See also Legend to Figure 3.

In the analysis of RT genes expressed in particular tissues (47 datasets) we obtained 337 significant motif peaks at p-value <= 10^-5^: 169 with TRANSFAC matrices, 18 with JASPAR CORE matrices, 48 with JASPAR phyloFACTS matrices and, 102 with 6mers (Additional data files 3, 4, 5, 6). Many of the motifs corresponded to common transcription factor binding sites, already detected in the analysis of all genes. To identify motifs that were directly related to tissue-specificity, we obtained a list of motifs that were significant in RT genes expressed in a given tissue but not in HK genes. We identified 58 different ones, found in one or a few related tissues. Of these, 14 corresponded to TRANSFAC matrices, 2 to JASPAR CORE matrices, 10 to JASPAR phyloFACTS matrices and, 32 to 6mers. Figure 4 shows a selection of such motifs. A number of them are well-known tissue-specific motifs. For example in genes expressed in liver, aside from the more general TATA and CAAT sites, there were significant peaks for HNF-1 (maximum peak at -79 with matrix V$HNF1_01), and HNF-4 (maximum peak at -92 with V$HNF4_01_B). HNF-1 and HNF-4 are members of the hepatocyte nuclear factor (HNF) family, and are well-known regulators of expression in liver and other related tissues [27]. Accordingly, the HNF-4 motif was also found in large intestine (main peak at -82 with V$DR1_Q3), and, with p-value <= 10^-3^, in small intestine (main peak at -78 with V$HNF4_01_B) and kidney (main peak at -91 with V$HNF4_DR1_Q3). The HNF-1 motif was also significant, at p-value <= 10^-3^, in kidney (maximum peak at -70 with V$HNF1_Q6).

Several motifs were repeatedly found in tissues from the nervous system (cluster A, Additional file 7). GC-box/SP1 and alphaCP1 motifs were particularly strong in nervous tissue genes. Among tissue-specific motifs, MZF1 was significant in cortex, hindbrain and midbrain (maximum peak between -39 to -44, p-value <= 10^-3^); AP2 in brain, cortex, hindbrain and striatum (maximum peak between -50 and -58, p-value <= 10^-3 ^in the three latter tissues); and EGR in striatum (maximum peak at -81, p-value = 6.08 × 10^-4^). There is evidence that the factors EGR1, AP2 and SP1 are required for the neuroendocrine-specific expression of chromogranine B gene [28]. Myeloid zinc finger 1 (MZF-1) is known to play a major role in myeloid cell differentiation. The enrichment we find in neural tissue expressed genes may mean that this factor regulates neural processes as well, or that the motif resembles the consensus sequences for another, yet uncharacterized, neural factor.

In testis, the RFX1 motif was significant (max. peak at -16 with V$RFX1_02), which is consistent with the abundance of RFX factors in this tissue [29]. This motif was not found in any other tissue. Similarly, MYB and PBX1 were only found only in bone marrow (max. peak at -4 and -473, respectively, p-value <= 10^-3^). MYB is known to be important for the regulation of hematopoiesis [30].

Interestingly, there were several tissue-specific motifs that could be detected with JASPAR phyloFACTS, or by 6mers, but not using matrices for known transcription factor binding sites. Many of these motifs are likely to correspond to yet uncharacterized transcription factor binding sites. For example phyloFACTs motif CTGCAGY showed a significant peak at +31 in brain, RGAGGAARY at -34 in bladder, and YCATTAA at -307 in mammary gland (Figure 4). Other putative tissue-specific motifs were only detected with 6mers. Examples include TAGAAC, at -353 in bone marrow, ATGAGA at -413 in hindbrain, and AGATTC at -490 in uterus.

### Transcription factor target predictions

An important outcome of this work was the prediction of many novel potential transcription factor sequence targets in the regions showing significant positional bias (p-value <= 10^-5^). It is a well-known fact that predictions of regulatory motifs suffer from the problem of false positive detection. However, given the strong position-dependency of the motifs found by PEAKS, predictions within the identified significant regions are expected to be much more reliable than predictions elsewhere in the promoter (see also next section). Using TRANSFAC matrices, predictions for commonly found binding sites (those in Figure 2A) were mapped to 5,798 different promoters (Additional file 8). This means that the vast majority of promoters (91%) contain at least one of the general regulatory motifs in the significant sequence range. Besides, we also obtained 559 predictions for motifs not significant in the ALL or HK datasets, providing annotations for putative tissue-specific transcription factor binding sites in 394 different promoters (Additional file 9). The total number of genes with one or more predicted motifs was 5942 (Additional file 10). Among tissue-specific motifs we found 86 RFX1 matches in 74 different promoters, 61 AP2 matches in 47 promoters, 40 PU1 matches in 34 promoters and, 32 HNF4 matches in 20 promoters.

### Comparison with experimental data

In a previous study using yeast promoters, we showed that regions identified by PEAKS were significantly enriched in real binding sites [14]. To compare the computational results of this study with experimental data, we systematically search all the experimental binding site annotations for mouse genes in TRANSFAC, and map them onto our genes. We recovered 35 non-redundant experimentally validated sites that could be successfully mapped to genes in our dataset, for GC-box/SP1, CAAT-box/NF-Y, CREB/ATF, YY1/NF-E1, GAB/ETS and HNF-4. In general, the computational and experimental results were in very good agreement, and 25 of the 35 sites fell within significant regions (p-value <= 10^-5^). By individual motifs, 15/18 of the GC-box/SP1 experimental sites, 5/7 of the experimental YY1/NF-E1 sites and 3/4 of the experimental CAAT-box/NF-Y fell within regions that were significant in the PEAKS analysis. For CREB/ATF, instead, only 1 out of 4 sites were located in PEAKS significant regions. For GABP/ETS and HNF-4 we only had one experimental site to compare with. The GABP/ETS site fell within the significant region. However, the HNF-4 site, in cytochrome P450 Cyp3a16, was located upstream from the region identified by PEAKS. This finding prompted us to scrutinize all other HNF-1 and HNF-4 experimental sites in TRANSFAC mouse gene entries, even if the genes were not in our dataset. These motifs were present in four additional TRANSFAC mouse gene entries: albumin 1 (HNF-1), alpha-fetoprotein (HNF-1 and HNF-4), retinol-binding protein II (HNF-4) and, transthyretin (HNF4). Of these 5 cases, 4 fell within the regions identified by PEAKS (-92 to -79), and only the HNF-4 motif in retinol-binding protein II was outside the significant region. In the work presented here, we found 31 additional putative HNF-4 sites, in different mouse promoters, which fell within the significant region. Given the positive outcome of the comparison between computational and experimental site locations, many of these sites are likely to be functional. In support of this, a region in which we predict HNF-4 sites in the hepatic lipase gene, has been recently observed to be responsible for enhanced promoter activity in liver cells, and for silencing expression in non-liver cells [31].

## Discussion

Important information on the spatio-temporal expression pattern of a gene is encrypted in gene promoter sequences. Within promoters, particular arrangements of regulatory motifs facilitate specific transcription factor interactions, which result in transcription activation or repression. Transcription regulatory regions can evolve quickly, and similar motifs are often present in genes with coordinated gene expression, even if the genes are not homologues. Recurrent motif arrangements are thus presumably the result of similar evolutionary constraints in genes that are part of the same regulatory network. In the present study we have focused on motif arrangement in the proximal promoter, using the distance from the transcription start site. Until now, studies on positionally biased regulatory motifs had only been performed for general promoter motifs [6,7,32], or, at the other extreme, for motifs found in very specific datasets of functionally related genes [10,33]. Here we have investigated the impact of motifs with positional bias in the configuration of promoters driving expression in various body tissues, and used this property to uncover potentially novel tissue-specific regulators.

A number of computational studies have established that particular DNA words tend to cluster in the vicinity of the transcription start site in mammalian gene promoters [6,7,32]. Our analysis indicates that the TATA box is not a particularly common motif, the peak observed using TRANSFAC matrix V$MTATA_B corresponds to only 3,4% of the genes, although given that this refers to a region +/- 15 bp of position -41, it is likely to be an under-estimation of the real number of sites. The low frequency of this motif is in strong contrast with previous ideas on the central role of this motif in transcription, but more in line with more recent estimates based on larger datasets [34,35]. Indeed, TATA-containing promoters are more typical of tissue-specific genes than of housekeeping genes, and show a high degree of conservation across species [34]. Promoters containing GC-rich SP1 binding sites, on the contrary, appear to be very widespread, and their frequency is higher in housekeeping than in tissue-specific genes (Figure 3). Other very common motifs in mammalian promoters include binding sites for the ETS family of transcription factors, for E2F1, and CAAT-box/NF-Y motifs. None of the known basic motifs in the core promoter appears to be universal, and each one is present in only a fraction of genes. Basic motifs can combine in different ways, and it has been shown that some combinations – such as CAAT and SP1 sites – are particularly common [6]. Interactions between several of the transcription factors that assemble at the core promoter have been described, including YY1 and SP1 [36], E2F and SP1 [37] and, NF-Y (CAAT-box) and TATA binding protein (TBP)-associated factors [38]. These protein interactions are likely to impose constraints on the relative positions of the corresponding DNA motifs, which would explain why we find such strong motif positional dependencies. In support of this, it has been shown that the activity of the thymidine kinase promoter depends on the distance between E2F motifs and upstream SP1 binding sites [37].

Our results strongly indicate that housekeeping gene promoters have more fixed promoter structures than the class composed of promoters driving restricted tissue expression. This is not surprising, as distinct regulators are expected to control expression in different tissue types. On the other hand, we have shown that the Ying and Yang (YY) downstream motif is a very important constitutive element of genes with broad expression, whereas it appears to be of little relevance in genes that show tissue-specific expression. Other motifs, such as E2F and CREB/ATF/E4F, also show much stronger peaks in housekeeping genes than in tissue-restricted genes. Interestingly, in the latter the maximum peak position is displaced towards a more upstream position (-59 in RT, versus -42 in HK, for E4F, Figure 3), pointing to possible mechanistic differences in the way these factors interact with the initiation complex in the two classes of genes.

The control of tissue-specific expression is still poorly understood. We have been able to identify a number of motifs that show positional bias in tissue-restricted datasets. Previous studies on the identification of tissue-specific motifs were based on cross-species conservation and subsequent detection of tissue enrichment [5], or on the identification of *cis*-regulatory modules with high tissue-specific expression predictive value [39]. In relation to the latter study, Smith et al. [40] provided a list of tissue-specific expression important motifs: HNF-1, HNF-3, HNF-4, C/EBP and DBP in liver; MEF-2, SRF, Myogenin and SP1 in skeletal muscle and; SRY, CREM, RFX in testis (see Table III of [40]). Of these motifs, we found that HNF-1 and HNF-4 in liver, and RFX in testis, showed significant positional biases. Instead, the above-mentioned muscle-specific motifs were not identified in our analysis. This could be due to a more flexible and variable arrangement of motifs in these genes, or simply to the motifs being outside the region of the promoter considered (proximal region). In relation to this, it has been recently proposed that motifs bound by RFX factors are very abundant in conserved non-coding regions, scattered throughout the genome [41]. In another study [42], using cross-specific conservation criteria, it was found that AP-2, SP1 and EGR-1 were over-represented in neural tissues. AP-2 and EGR-1 showed positional bias in several nervous system tissues. On the other hand, SP1, while significant in the majority of tissues, achieved the largest positional footprinting scores in mammary gland, brown fat and pancreas.

Many of the motifs that show significant positional bias in our analysis are located within the first 100 bp upstream of the TSS. This is not surprising considering that the sequences are anchored at the TSS in this analysis, and position dependencies between interacting motif-binding proteins are expected to be more relevant for short distances [36,37]. More unexpected is the presence of motifs with positional bias much further upstream, in several tissue-restricted datasets. This includes MEIS1BHOXA9 in liver (maximum peak at -433), PBX1 in bone marrow (maximum peak at -473, p-value = 4.22 × 10^-4^), STAT5A in eye (maximum peak at -469, p-value = 4.26 × 10^-4^), and OCT1 in olfactory bulb (maximum peak at -540, p-value = 8.35 × 10^-4^). One possibility to explain these cases is the existence of stronger evolutionary constraints in a longer portion of the promoter. Our own data on the weaker sequence conservation of housekeeping promoters with respect to tissue-specific distal promoters, particularly upstream from position -500, points in this direction [43]. On the other hand, from this study it can also be concluded that, contrary to what is generally assumed, the motif content of the region around the TSS can vary greatly depending on specific tissue expression. Dataset-specific motifs with positional bias have also been identified in ribosomal gene [10] or histone-coding gene promoters [33]. Therefore, both shared motif content and shared relative motif positions appear to be important for the regulation of genes with similar tissue expression patterns.

## Conclusion

In this work we have shown that motifs with positional bias are abundant in mammalian promoters and can be used to define distinct promoter architectures depending on breadth or tissue of gene expression. The results offer new insights into the shaping of motif arrangement in promoter sequences by evolutionary processes. We provide predictions for a large number of motifs, including general as well as tissue-specific motifs, that show positional bias. This work provides a foundation for future studies on motif position constraints in gene regulatory sequences.

## Methods

### DNA sequences and tissue expression data

Gene datasets were defined from mouse transcriptome microarray data from Zhang et al. [16]. The corresponding gene promoter sequences were extracted from UCSC database (mm6) [17]. We selected genes that had a unique annotated TSS in the database as a representative set. The analysis comprised 6,372 non-redundant promoter sequences, which spanned from -600 to +100 relative to the TSS position. These sequences define the ALL dataset. Subsequently, genes were classified in 3 classes according to expression breadth: housekeeping (HK), 1,544 genes expressed in 51–55 tissues; intermediate, 3,006 genes expressed in 11–50 tissues and; restricted (RT), 1,822 genes with expression restricted to 1–10 tissues. Because many tissues can share cell types, or cell functions, we calculated the number of shared genes between tissues. We measured the overlap between all pairs of tissues and selected those pairs sharing at least 30% of genes. We selected 4 clusters that contained more than 2 adult mouse tissues. They showed a good agreement with physiological systems: 'nervous' (A), 'digestive/kidney' (B), muscular/skin' (C) and, 'skeletal/lymphatic/bladder' (D). They are shown in Additional file 7.

### DNA motif prediction

For the detection of known motifs in the sequences we used three weight matrix collections of transcription factor binding sites: TRANSFAC 7 containing 508 vertebrate position specific weight matrices (PSWMs), JASPAR containing 91 vertebrate CORE PSWMs and, JASPAR 174 phyloFACTS PSWMs. Sequence hits to a matrix were defined as those that showed an overall matrix relative similarity score >= 0.90 and, for TRANSFAC matrices, an overall matrix relative similarity score >= 0.85 and core similarity score >= 0.99 [18]. To measure similarity to the TRANSFAC matrices we implemented the metrics described in [44], as used in the program MatInspector. For JASPAR matrices we used log-likelihood ratio scores. We also scanned the sequences for perfect matches to all oligomers of size 6 (6mers). Matches to both the sense and the anti-sense strand were considered. For this reason, the number of effective 6mers to be tested could be reduced from 4096 to 2080 (including 64 palindromic 6mers).

### Positional footprinting (PEAKS analysis)

For those DNA motifs that showed at least one match in any promoter sequence we performed PEAKS analysis. In this analysis, all sequences were of the same length (*l*) and contained a common element, the transcription start site (TSS), used as the reference position. For each DNA motif we scanned the sequences with a sliding window (*w*, uneven size) and counted the number of sequences that contained at least one occurrence of the DNA motif (motifs were matches to PSWMs or 6mer, see above) within that window, assigning this number, *n*(*i*), to the window central nucleotide, *i*. We used these values to build a motif profile along the sequence positions. In order to determine the positional bias of a motif we assigned a signal to noise score to each profile and estimated its p-value using random sequence datasets (see below). We then extracted the significant positions where the motif was located.

To measure the positional bias of a motif, which is basically the number of motif occurrences, at a particular position, *n*(*i*) relative to the background signal level, we use the positional footprinting score *Spf *[14]. It results from three diverse scores. The first score (*Sn*) measures the number of motif occurrences at a specific position with respect to the average number along the sequence. The second score (*Sr*) penalizes signals present in only a very small percentage of sequences, by dividing the number of ocurrences at the specific location by the number of sequences used. Finally, the third score (*Sm*) is the number of occurrences at that position divided by the total number of motif predictions, used to penalize matrices that are very noisy and occur at a very high frequency, which is often due to low specificity of the matrix. As the scores account for different aspects of the signal to noise ratio, we multiply them to obtain a single final score: *Spf *= *Sn Sr Sm*. See PEAKS web documentation for a more detailed description of the *Spf *score [15].

The maximum value of the positional footprinting score *Spf*, which corresponds to the maximum peak of the motif, was defined as *Spf *_max. To assess the significance of *Spf *_max for each DNA motif tested in the dataset, we used 1000 different synthetic sequence datasets (see below). In each simulation we kept the random *Spf *_max. We then counted how many simulations showed a random *Spf *_max equal or higher than the observed *Spf *_max, and obtained the corresponding empiric p-value. The *Spf *_max values were distributed according to an extreme value distribution. We used this property to estimate the p-value that corresponded to a given score using linear interpolation. After selection of a p-value cut-off, the significant regions were defined by the concatenation of all positions that showed a score associated with a p-value below the cut-off.

Throughout this study we used *w *= 31 and p-value <= 10^-5^, unless stated otherwise. In addition, we filtered those motifs that, even if statistically significant, showed multiple peaks or very weak peaks (less than two fold motif frequency at the maximum peak position with respect to the background or *Spf *_max < 15).

### Construction of synthetic datasets

In the synthetic datasets, each random sequence had a similar composition than a real sequence in the dataset. This was achieved by using three order 1 Markov models, each of which corresponded to a compositionally different region. The three compositionally different regions were defined in the complete mouse promoter sequence dataset. The first type corresponded to CpG islands, regions of length at least 200 bp, with a minimum GC content of 55% and a minimum observed/expected GpG content ratio of 0.65 [43]. The second type corresponded to GC-rich regions that did not conform to the CpG island definition. They were at least 200 bp long and had a minimum GC content of 55%. The remaining regions made the third type. Each promoter in the study was partitioned into these three regions. Of course, different promoters varied in the number and extension of these regions. We then concatenated all the regions that were of the same type to construct three different order 1 Markov chains. Each random sequence was generated using one, two or three Markov chains, preserving the partitioning in different regions observed in the original sequence. By this approach, we obtain synthetic datasets that were remarkably similar in composition to real datasets along the promoter (Additional file 1).

### Motif clustering

There was a considerable amount of redundancy in the motifs identified, both within and across motif libraries. To disentangle it, we clustered the motifs using hierarchical clustering (R package complete hierarchical clustering, [45]). Distance between motifs was based on the proportion of overlapping motif matches along all non-redundant promoter sequences. Specifically, say we have motif A and motif B (represented as matches to PSWMs or 6mers). Then the distance between A and B will the dist (*A*, *B*) = ((*N*(*A*, *B*)/*N*(*A*))+(*N*(*A*, *B*)/*N*(*B*))/2, where *N*(*A*, *B*) is the number of predictions of motif A and predictions of motif B that overlap, *N*(*A*) the total number of predictions of motif A and, *N*(*B*) the total number of predictions of motif B. The dissimilarity cut-off used was 0.98. This approach resulted in 9 different clusters out of a total of 65 significant motifs in the complete mouse promoter dataset (Additional file 2).

### Mapping of significant motifs in promoter sequences

The PEAKS analysis yielded significant regions for various motifs in each of the datasets tested. We extracted the actual predictions of the motifs in the promoter sequences, considering those motifs that fell within the significant region, and those located up to 15 nucleotides upstream or downstream of this region, as they also contributed to the peak considering that the window size employed was 31. Additional files 8 and 9 contain the predictions of general (significant in the complete collection of mouse promoters) as well as non-general motifs. Additional file 10 is a zipped file containing individual files with predictions of general and non-general motifs per each gene, in BED format. This includes 5942 genes, for which we found significant motif predictions, and a README file with instructions on how to visualize them using UCSC Genome Browser.

### Global over-representation statistics

We calculated motif frequencies in complete promoter sequences using the PSWM predictions as described previously. To assess if a motif was over-represented in a particular dataset we calculated the corresponding p-value using synthetic datasets as described for positional footprinting. In addition, we compared the relative abundance of the motif in the particular dataset to that obtained in the general dataset (ALL). The values for each motif that showed significant positional bias are provided in Additional files 3, 4, 5, 6.

## Authors' contributions

NB and MMA designed the study. NB and DF carried out the computations. NB, DF and MMA analyzed the data. NB and MMA wrote the manuscript. All authors read and approved the final manuscript.

## Supplementary Material

Additional file 1 Additional file 1 contains average nucleotide composition along the promoter for real and synthetic datasets. Click here for file

Additional file 2 Additional file 2 contains motif clustering for motifs in Figure 2. Click here for file

Additional file 3 Additional file 3 contains results of motif positional bias searches using TRANFAC matrices, at p-value <= 10^-5^. Click here for file

Additional file 4 Additional file 4 contains results of motif positional bias searches using JASPAR CORE matrices, at p-value <= 10^-5^. Click here for file

Additional file 5 Additional file 5 contains results of motif positional bias searches using JASPAR phyloFACTS matrices, at p-value <= 10^-5^. Click here for file

Additional file 6 Additional file 6 contains results of motif positional bias searches using 6mers, at p-value <= 10^-5^. Click here for file

Additional file 7 Additional file 7 contains tissue clusters in which every pair of tissues shares at least 30% of the genes of restricted (RT) expression class. Click here for file

Additional file 8 Additional file 8 contains predictions of general motifs in mouse promoters, in significant regions described in Additional file 3. Click here for file

Additional file 9 Additional file 9 contains predictions of non-general motifs in mouse promoters, in significant regions described in Additional file 3. Click here for file

Additional file 10 Additional file 10 contains the data in Additional files 8 and 9 in BED format, for visualization using UCSC Genome Browser. Click here for file
