# Supplementary material for: Positional bias of general and tissue-specific regulatory motifs in mouse gene promoters
Source: BMC Genomics. 2007 Dec 13;8:459. doi: 10.1186/1471-2164-8-459 (PMC2249607; doi:10.1186/1471-2164-8-459)
Supplement: Additional file 1 — contains average nucleotide composition along the promoter for real and synthetic datasets. [file 1471-2164-8-459-S1.pdf]

Figure 1 - Background model

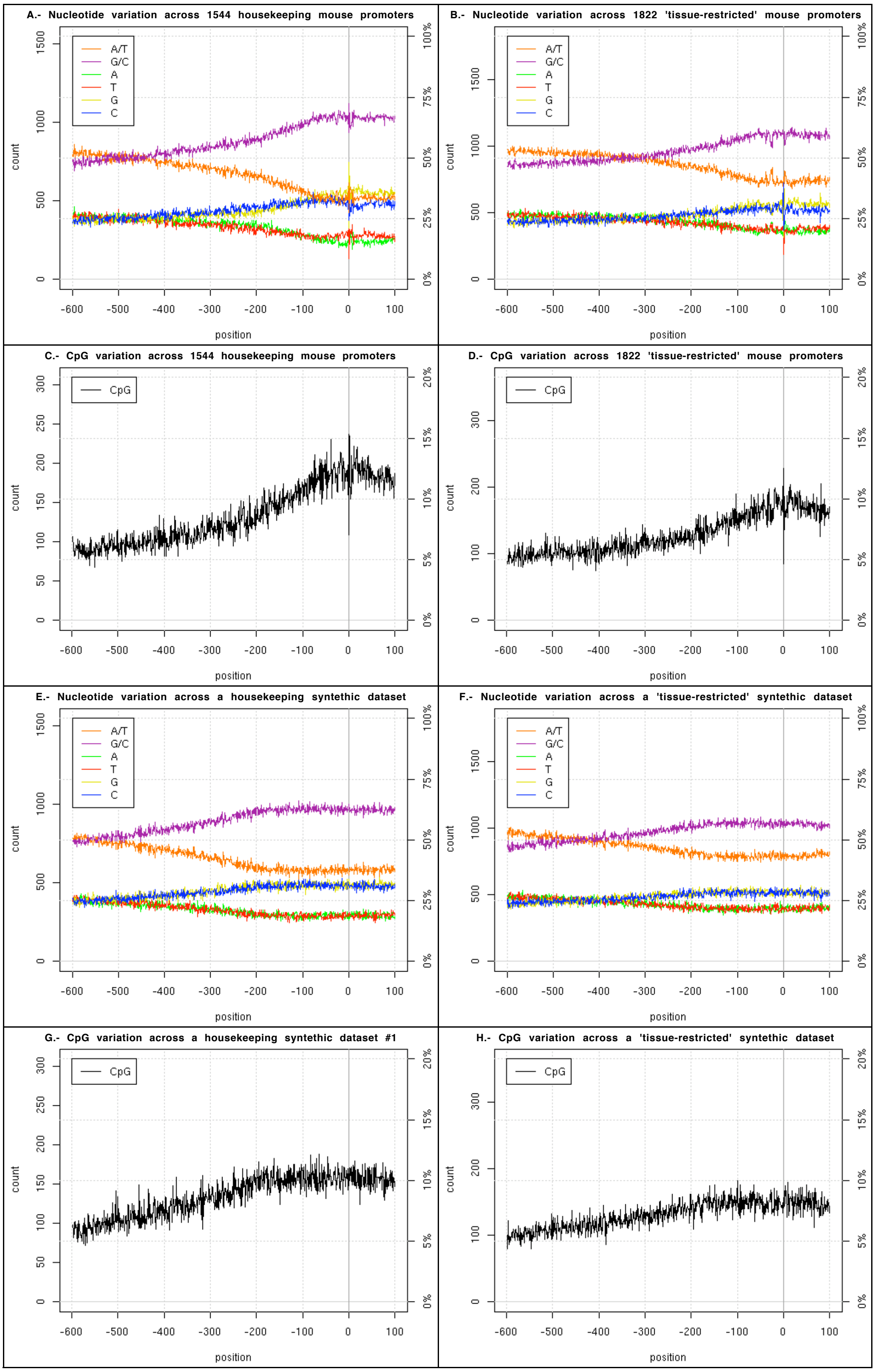

Figure 1 Background model **A, B, C, D** Real variation across mouse housekeeping and 'tissue restricted' promoters of mono-nucleotides and CpG dinucleotide. **E, F, G ,H** Nucleotide and CpG variation across sequences on synthetic datasets.
