# Supplementary material for: Positional bias of general and tissue-specific regulatory motifs in mouse gene promoters
Source: BMC Genomics. 2007 Dec 13;8:459. doi: 10.1186/1471-2164-8-459 (PMC2249607; doi:10.1186/1471-2164-8-459)
Supplement: Additional file 2 — contains motif clustering for motifs in Figure 2. [file 1471-2164-8-459-S2.pdf]

Figure 2 - Motifs clustering

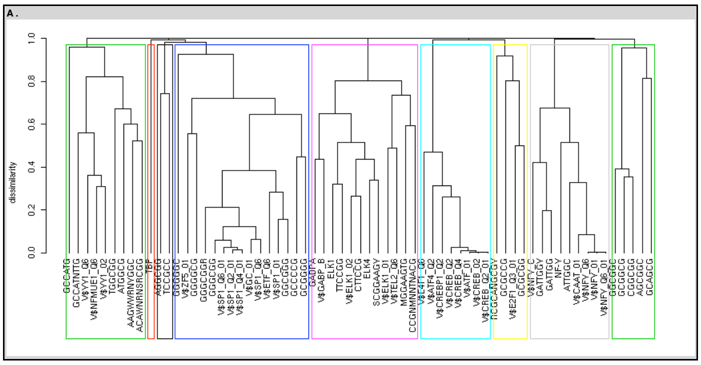

B .

1	CLUSTER_1	
1	AAGWWRNYGGC	JASPAR_PHYLOFACTS
2	ACAWNRNSRCGG	JASPAR_PHYLOFACTS
3	GCCATNTTG	JASPAR_PHYLOFACTS
4	V$NFMUE1_Q6	Transfac 7
5	V$YY1_02	Transfac 7
6	V$YY1_Q6	Transfac 7
7	TGGCGG	6-mer
8	ATGGCG	6-mer
9	GCCATG	6-mer
2	CLUSTER_2	
1	TBP	JASPAR_CORE
3	CLUSTER_3	
1	AGGCGG	6-mer
2	TCCGCC	6-mer
4	CLUSTER_4	
1	GGGCGGR	JASPAR_PHYLOFACTS
2	V$ETF_Q6	Transfac 7
3	V$GC_01	Transfac 7
4	V$SP1_01	Transfac 7
5	V$SP1_Q2_01	Transfac 7
6	V$SP1_Q4_01	Transfac 7
7	V$SP1_Q6	Transfac 7
8	V$SP1_Q6_01	Transfac 7
9	V$ZF5_01	Transfac 7
10	GCCCCG	6-mer
11	GGGGGC	6-mer
12	GCGGGG	6-mer
13	GGGGCG	6-mer
14	GGCGGG	6-mer
15	GGGCGG	6-mer
5	CLUSTER_5	
1	GABPA	JASPAR_CORE
2	ELK1	JASPAR_CORE
3	ELK4	JASPAR_CORE
4	SCGGAAGY	JASPAR_PHYLOFACTS
5	MGGAAGTG	JASPAR_PHYLOFACTS
6	CCGNMNNTNACG	JASPAR_PHYLOFACTS
7	V$ELK1_01	Transfac 7
8	V$ELK1_02	Transfac 7
9	V$GABP_B	Transfac 7
10	V$TEL2_Q6	Transfac 7
11	CTTCCG	6-mer
12	TTCCGG	6-mer
6	CLUSTER_6	
1	V$ATF_01	Transfac 7
2	V$ATF4_Q2	Transfac 7
3	V$CREB_02	Transfac 7
4	V$CREBP1_Q2	Transfac 7
5	V$CREB_Q2	Transfac 7
6	V$CREB_Q2_01	Transfac 7
7	V$CREB_Q4	Transfac 7
8	V$E4F1_Q6	Transfac 7
7	CLUSTER_7	
1	RCGCANGCGY	JASPAR_PHYLOFACTS
2	V$E2F1_Q3_01	Transfac 7
3	GCGCGG	6-mer
4	GCGCCG	6-mer
8	CLUSTER_8	
1	NF-Y	JASPAR_CORE
2	GATTGGY	JASPAR_PHYLOFACTS
3	V$CAAT_01	Transfac 7
4	V$NFY_01	Transfac 7
5	V$NFY_C	Transfac 7
6	V$NFY_Q6	Transfac 7
7	V$NFY_Q6_01	Transfac 7
8	GATTGG	6-mer
9	ATTGGC	6-mer
9	CLUSTER_9	
1	AGCGGC	6-mer
2	GCGGCG	6-mer
3	CGGCGG	6-mer
4	GCAGCG	6-mer
5	GGCGGC	6-mer

Figure 2 Motifs clustering. **A** Clustering of 65 significant motifs on ALL dataset. Distance between motifs based on overlaping matches between motifs. Hierarchical cluster analysis using R, method = 'complete', dissimilarity cut-off = '0.98'. **B** 65 significant motifs from transfac, jaspar_core, jaspar_phylofacts and hexamers were clustered into 9 groups.
